# Chinese language teachers’ dichotomous identities when teaching ingroup and outgroup students

**DOI:** 10.3389/fpsyg.2022.939333

**Published:** 2022-07-29

**Authors:** Haijiao Chen, Wanting Sun, Jinghe Han, Qiaoyun Liu

**Affiliations:** ^1^Department of Faculty Development, Huaqiao University, Xiamen, China; ^2^School of Education, Western Sydney University, Sydney, NSW, Australia; ^3^Department of Ideology and Politics, Guangxi Art School, Guangxi, China

**Keywords:** language teacher identity, Chinese language teacher, contextualized social processes, multiculturalism, self-conceptualizations

## Abstract

Research into second language teacher identity has experienced a shift in recent years from a cognitive perspective to social constructionist orientation. The existing research in Chinese language literature in relation to Foreign Language (CFL) teachers’ identity shift is principally in relation to the change of social, cultural, and institutional contexts. Built on the current literature, this research asks: “How might teachers’ self-images or self-conceptualizations be renegotiated when they are located within their own mainstream cultural and educational system, yet comprised of students from various cultural backgrounds?” The data were collected from a group of CFL teachers in a South China university. The research found that students’ backgrounds largely impacted on, and led to, the teachers’ dichotomous relational identities, but did not dramatically change the teachers’ perception on what or how much subject knowledge to be possessed to make an ideal CFL teacher. This attribute of their identity was sustained even though the teaching content was modified at a practical level in response to groups’ differences. Further, the CFL teachers’ pedagogical identity remained stable with only minor modifications when teaching “ingroups” and “outgroups” of students.

## Research background

The last two decades has witnessed a growing body of literature on the topic of language teacher identity due to a proliferation of diverse roles in (language) teaching. Researchers acknowledge that understanding language teachers’ identities is vital for initial teacher education and teachers’ further professional development (Beauchamp and Thomas, 2009; Huang, 2019). Language teacher identity plays a foundational role in guiding teachers’ recursive construction and reconstruction of knowledge and competence (Morgan and Clarke, 2011). It is essential in teachers’ adaptation to the dynamic and evolving language education environments (Tao and Gao, 2018). Teacher identity helps language teachers to appreciate what constitutes a good teacher, teachers’ subject knowledge, and pedagogical knowledge (Johnston et al., 2005). A professional self-image can optimize teachers’ functionality within their working environment (Yuan and Burns, 2017), and eventually achieve quality language teaching (Wang and Du, 2016).

Nevertheless, research into second language teacher identity has experienced a shift in recent years from cognitive perspective to social constructionist orientation (Pennington and Richards, 2016; Yazan, 2018). This is a shift of focus to the examination of teachers’ identity in contextualized social perspectives, in contrast to the earlier effort on psychological practices and influences (Miller, 2009). Theorists in the field hold the belief that teacher identity is not fixed nor is it imposed; it is a context-bound, fluid phenomenon (Varghese et al., 2005), and “negotiated through experience” (Sachs, 2005, p. 15). The contexts range from the macro-levels of social, cultural, and political milieu to immediate micro-spaces where teaching occurs. Teachers’ “identity emerges as a dynamic construct that is shaped by the context in which the teacher works” (Pennington and Richards, 2016, p. 6). However, the mechanics of how teacher identity is shaped by contexts needs more attention.

The existing research in Chinese as Foreign Language (CFL) teachers’ identity is principally focused on the changes in social and cultural contexts. Reports are around teachers moving within and between environments, such as social, cultural and institutional contexts. For example, an investigation on teachers’ identity found pre-service teachers shaped their identity differently in macro-distinctive social structures of Hong Kong and Mainland China (Gu and Benson, 2015). Another research found Chinese language teachers’ professional identities “co-existed” and overlapped with their social and cultural identities in mediating intercultural communicative competence in Hong Kong’s international schools (Gong et al., 2021, p. 6). Studies conducted with expatriate Chinese teachers in the “new” social and cultural contexts such as the United States, the United Kingdom, and Australia, found their professional identities experienced a renegotiation process cognitively and emotionally in accordance with their social relations and cultural practices (Yang, 2019). Their adaptation also included a pedagogical shift to cope with the needs of local school systems and students (Zhang, 2015; Xiang, 2021). The studies detailed a linear relation between identity and context. They ascribed teachers’ identity shifts to the change of macro contexts, but mostly left the contexts unspecified. This approach left the question unanswered: “Which particular context – social, cultural or institutional – or all, had inevitable impact on which aspect of teacher identity?

Students as a key and immediate “context” have been studied extensively but how their ethnic and cultural background might be a distinguished variable in relating to teachers’ identity development or shift is yet to attract similar attention. There is a plethora of studies focusing on how students in general impact on teachers’ relational identity. One study found that teachers’ emotions were related to the students’ behavior and performance outcomes (Day and Kington, 2008). The authors posited that teachers’ emotions were affected by their “immediate working context” and were “connected to (their) long-term identity” (Day and Kington, 2008, p. 1). One study explored the correlation between teachers’ identity and students’ learning (Ben-Peretz et al., 2003) and found that teachers’ professional images of self were closely allied to their students’ achievements. In this research, students were presumed to be one generic group, with only an implicit categorization as “good” and “bad” students based on their behavior and performance, impacting on teachers’ emotional identity. Nevertheless, a few studies have given attention to teachers’ identity and their cultural mismatch with their students’. For example, Zembylas (2010) centered on teachers’ emotional experiences, claiming that they demonstrated discomforting emotions in ethnically diverse and multicultural schools. It reveals that teachers’ emotional identity was impacted by the students’ “diversity” and the schools’ “multiculturalism” (p. 713). Johnson (2003) in her research confirmed that cross-cultural teacher-student interactions occasionally moved beyond the classroom walls, and teachers’ personal selves sometimes had an influence on their professional relationships with students.

There are some insightful studies on students’ impact on teachers’ pedagogical identity which are enlightening for this research undertaken. For example, findings in Gao’s (2012) study implied that CSL teachers in Hong Kong see themselves as “linguistic torchbearer” and “cultural transmitter” in relation to South Asian learners. Chinese teachers shifted pedagogy from an authoritative, parental “subject expert” to a plain and equal “culture worker and a learning facilitator” to cater for the language learners in Denmark (Wang and Du, 2014, p. 429). This research indicated that the cultural background of students played an agential role in crafting teacher identities. However, given the change of the physical environment where teachers were situated in teaching, it is not clear whether the pedagogical shift was completely due to the new students or because of other factors in the new macro teaching context. A more informative study conducted among CSL university teachers in China found that when teaching international students, CSL teachers were more skill-oriented than knowledge-oriented, and their perception of teaching was related to context factors (Gong et al., 2018). This research indicates that even when the social, cultural, and institutional context of teaching remained stable, the pedagogical identity may still shift in some extent if their students’ culture was mismatched with that of the teacher. This indicates that students’ ethnic and cultural background may be more powerful in teacher identity development than the teachers’ general social, cultural or institutional circumstance.

The gaps in current research in the area of dynamic CFL teacher identity are evident. Firstly, the student cohort as an immediate context that impacts on teacher identity was often not distinguished from other contextual factors (e.g., institutions). Mainstream studies on CFL teachers’ identity typically occurred in a new country, a new school and with new students, and thus “what influences what” is less clear. Secondly, the impact on teachers’ identity from the student groups that shared cultural similarities (ingroup) and culturally mismatches (outgroup) with the teachers were often not differentiated in a comparative manner. The literature revealed that students from other cultural backgrounds impacted on the teachers’ emotional or pedagogical shift or adjustment, but how ingroup and outgroup students may differently influence teachers is yet to be extensively studied. Built on the current literature, this research asks: “How might teachers’ self-images or self-conceptualizations be renegotiated when located within their own mainstream educational system and yet comprised of students from various cultural backgrounds?” It explores teachers’ understanding of their subject knowledge in terms of what a CFL teacher should know and teach, and how their pedagogical and relational identity varies or shifts when engaging with different cultural groups. The comparative approach of this current research is based on the teaching of local and international cohorts within the overall stable cultural and institutional macro teaching context: that is, within the teachers’ home environment.

## Theoretical framework

This research draws on the analytical tools provided by Social Identity Theory (Hogg, 2018) from the perspective of modernism; and the post-modern notion of Multiple Identities Approach (Gee, 2000). The combination of these two theories targets an exploration of the dichotomy between an unremitting fluidity and temporary stability of identity. It investigates: the contextual community’s impact on the construction and reconstruction of teachers’ identities, the influences on the teachers’ dynamic moments through their conceptualization of self and examines the impact of the cultural “ingroup” as distinct from the “others” – other cultural groups.

Social Identity Theory addresses “collective phenomena” that “cannot be adequately explained in terms of isolated individual processes or interpersonal interaction alone” (Hogg, 2018, p. 112). It addresses issues such as “intergroup conflict, conformity, normative behavior, group polarization, and crowd behavior” (Hogg, 2018, p. 112). Self-categorization is the cognitive dimension of the theory informing the notion of “us”. It describes how categorization of self and others underpins social identification and associated group and intergroup phenomena (Hogg, 2018). McLeod (2008) classifies this mental process into three stages: categorization, identification, and comparison. The first stage draws on social and cultural knowledge to categories people. These categories include gender, race and profession, such as black or white, Chinese or American, and student or teacher. These aspects are believed to have Psychological Salience and affect behavior, thus are valued and frequently employed in self-conceptualization (Hogg, 2018). The second stage is social identification, involving the allocation of the self into the stereotypical groups categorized at stage one. Categorized groups are identified with different sets of “attributes” or prototypes, such as perceptions, attitudes, feelings, and behaviors (Hogg, 2018, p. 119). Group members tend to share similarities within and across these aspects and act according to their imagined ingroup attributes. The final stage involves social comparison between ingroup and outgroups (McLeod, 2008). The comparisons are oriented toward similarity, assimilation, and uniformity as it is argued that “social comparisons between groups are focused on the establishment of distinctiveness between groups” (Tajfel, 1972, p. 296). Social Identity Theory in this research provides a framework to understand how the Chinese CFL teachers see themselves culturally or ethnically similar to their local students; or different from their students coming from other ethnic backgrounds. These perceptions then shape how they position and reposition themselves in teaching.

The limitation of Social Identity Theory is its dependence on an “individual’s knowledge” of categorizing social groups (Tajfel, 1972, p. 292), drawn from their experiences with “readily accessible social categorizations” such as race, gender, and profession (Hogg, 2018, p. 121). This may not then take account of individuals’ knowledge and functioning in different contexts. To supplement this, the Multiple Identity Approach is useful. This post-modern theory acknowledges “‘core identity’ that holds more uniformly, for ourselves and others, across contexts” (Gee, 2000, p. 100). Concurrently it posits that “all people have multiple identities” and they are “connected to their performances in society” (Gee, 2000, p. 100). It criticizes the “overly general and static trio of ‘race, class, and gender”’ of modernism, and endorses the idea that “when any human being acts and interacts in a given context, others recognize that person as acting and interacting as a certain ‘kind of person’ or even as several different ‘kinds’ at once” (Gee, 2000, p. 99). The Multiple Identity Approach heeds the dynamic side of identity especially in contextually specific situations. Thus, this theory provides a conceptual framework for examining the current research undertaken, in terms of how the teachers see themselves differently or behave differently in different contexts especially when in front of students of different cultural groups. It opens up consideration of whether the participant Chinese teachers share teachers’ attributes with others in the world despite stereotypical notions of race or nationality.

The research undertaken is geographically situated in a metropolitan Chinese university. Through the lens of Social Identity Theory, the participant teachers’ self-imaging and self-conceptualization of their core identity as CFL teachers is expected to be captured through their narrated experiences. These teacher narratives highlight the attributes of self and/or their similar ingroup, and may originate from their accumulated prototypical, preconceived knowledge of teaching as a profession: potentially a static view of such sociocultural characteristics as the “linguistic, ethnic, racial, and gender” (Yazan, 2018, p. 27) of students and teachers. The teachers’ understanding of self-identity is articulated through their individual and professional identities as “pre-context” (in that they are pre-determined by their linguistic, ethnic, racial, and gender status), and thus may be temporarily stable. From the perspective of a Multiple Identity Approach, the CFL teachers in this research interact with their local ingroup Chinese students, who were similar linguistically, culturally and possibly ethnically. At the same time, included in their classrooms are students from overseas, termed “outgroup” in this research. Contextually specific analysis of teachers’ interview data captures the fluidity of teacher identities in response to the change responses in and across “mini-contexts”. Through this lens the teachers’ identity negotiations and switching in response to their active participation in various cultural groups of teaching and learning is apprehensible. In this way the use of the two conceptual frameworks of Social Identity Theory and Multiple Identity Approach enables a more complex and nuanced understanding of identity creation as being both stable and dynamic.

## The research

In this research, a qualitative approach underpinned by an interpretivist paradigm is employed to seek teachers’ views of themselves and their identity as teaching professionals. Interpretivism stresses the need to locate analysis in context, as individuals understand the external world from their subjective experiences (Reeves and Hedberg, 2003). The objective of this research is to explore the identity of Chinese background CFL teachers as they engage with students from various cultural groups within their daily practice. Adopting an interpretivist paradigm provides possibilities for understanding and theorizing the teachers’ identity through their subjective conception of self (Pennington and Richards, 2016).

The research was conducted at a university in Southern China. Six Chinese background CFL teachers were recruited from its Chinese Language and Culture College. The University was selected as the research site due to its CFL courses offered to both Chinese local and international students. With the assistance of the Academic Affairs Office recruiting documents were emailed to the faculty teachers who had had CFL teaching experiences with local and international students. Six teachers expressed interest and participated the interview (see Table 1).

**TABLE 1:** The participants’ background.

Fictious name	Gender	Age	Teaching experience	Teaching areas
Jing Lin	Female	33	7 years	Chinese lexicology
Wei Le	Female	30	4 years	Chinese linguistics
Wei Wang	Female	37	10 years	Chinese linguistics
Xia Xiao	Female	41	11 years	Chinese lexicology
Xing Liu	Female	33	4 years	Chinese poetry appreciation
Yuan Wang	Female	27	1 year	Culture of Chinese characters

Interviews were the primary source of data reported in this manuscript. A semi-structured interview protocol was employed to maintain the interview focus *via* a prepared set of guiding questions, whilst still permitting the expansion of the initial interview scope (Morse, 2012). The interview questions were designed to reveal the participants’ self-conception as teachers, particularly their teacher’s subject matter, relationships, and pedagogical identities when teaching students with the same, and different language and cultural backgrounds to their own. The interviews were conducted in Chinese language – the participants’ first language (L1), considering they were more comfortable with the language when expressing themselves (see “Appendix A” for the interview protocol). Authors 2 and 3 conducted the semi-structured interviews and Authors 1 and 4 manually transcribed the data. Author 2 translated the data into English, and to ensure the accuracy authors 1 and 3 checked through the translated documents. To keep the participants’ identity anonymous, pseudonyms were used in all the transcripts.

The data were scrutinized through thematic analysis, an umbrella term for a variety of approaches, rather than a singular method (Braun and Clarke, 2006; Guest et al., 2012). One of the traits of thematic analysis is its flexibility—flexibility with regards to framing theory, research questions, and research design (Clarke and Braun, 2017). As with most research methods, the process of data analysis in this research occurred in two primary ways—inductively and deductively (Braun and Clarke, 2006). Through an inductive process, the themes identified or generated were data driven. In applying deductive processes, the coding was directed or guided by the two reviewed theories. This process was more interpretative as the analysis was shaped and informed by reviewed and selected theories and concepts. Specifically, the process followed Braun and Clarke’s (2006) protocol: familiarizing with the data, open coding, highlighting, and collating initial codes, categorizing the codes with reference to the research questions, identifying themes, refining and interpreting themes, and theory-directed synthesis and development of arguments.

## Findings

Data reveal that the teachers’ self-conceptualization of identity is a result of the complexities between their pre-existing notions of “local” and “international” groups of students and their interactive experiences with each. They labeled the local as “our own students” whereas international students were referred to as “overseas” and “they”. This dichotomy resulted in an “ingroup” comprising the teachers and the local students and an “outgroup” encompassing the international “they” students, somewhat bundled together as a unified group regardless of their diversity across nationality, and ethnic and linguistic backgrounds. This division created the situation where the identity of the CFL teachers themselves operated across two junctures in relation to their understanding of CFL as a subject and its content knowledge, positioning self as a teacher when relating to students, and pedagogical adjustment when teaching the “ingroup” and the “outgroup”. These switches in identity were exemplified in practice and revealed that this phenomenon was not just a matter of individual identity adjustments. Data indicates that the teachers as a group demonstrated some common tendencies in identity shifts.

### Proud authoritative knowledge holders versus down-to-earth practical technicians

Based on the interview data, the teachers appeared to differentiate the two groups of students in terms of levels of expectations and responsibilities. Data reveal that for the student “ingroup”, content was expanded from a language-only focus to teaching language, culture, and history, and from direct language skills to an approach valuing language and theoretical understanding. The curricular and pedagogical content was thus expanded from language practice to teaching a comprehensive knowledge of the language system (Table 2). Evidence of variation in expectation and responsibilities for “ingroup” and “outgroup” persisted across the teacher interviews even though both groups were being educated to be future CFL teachers. One participant teacher acknowledged that “local and international students enrolled in different programs … all will be CFL teachers after graduation” (Jing Lin).

**TABLE 2:** Teacher as an academic knowledge holder or a down-to-earth practical technician.

Dichotomy	International – “outgroup”	Local – “ingroup”
Teacher’s understanding of TCFL as subject knowledge and teaching content	Language only (Hanzi) with minimum culture	Extension to Hanzi culture and history
	Language skills with basic level theoretical explanation	Extension to theoretical understanding
	Language application with limited theoretical knowledge	Extension to theoretical knowledge
	Specific practical skills	Teaching a complete knowledge system

The teachers’ expectations of local students are substantively different from expectations of the international cohort. The teachers mostly believe that their local students need a curriculum inclusive of more advanced knowledge beyond a limited language focus, featuring classic and/or contemporary pieces of literature, and providing an historical and cultural infusion into the subject knowledge:

For our own local students, my teaching is always linked to Chinese literature and history as we say language, literature and history are not separable. As a CFL teacher of Chinese background, they do need to have profound knowledge beyond the language itself (Xing Liu).
I need to give them (local students) a complete knowledge system. I must cover this amount of content within the semester (Wei Le).
I extended it (teaching and content) far more than the textbook. I use the textbook as a reference, but I like to add new theories and some advanced knowledge in the field (Xia Xiao).

Their understanding of suitable subject content for local students includes teaching “profound knowledge” (heritage knowledge) (Xing Liu), a “complete knowledge system” (Wei Le) and “advanced” knowledge (Xia Xiao). The teachers acknowledged that the selected content for the “ingroup” was broader and deeper. It should be beyond language and at knowledge level. Across this group of teachers, the international students received a tailored curriculum for their CFL course with a focus on language competence and skills, aiming at meaning making and communication at a practical level:

For international students, we have prescribed textbooks, which are quite scientifically designed. I basically follow the textbook and add some exercises and activities for them to practice (Jing Lin).
Since they are going to teach Chinese after graduation, I choose the words that they are more likely going to use or teach when they become teachers. The content is more practical (Yuan Wang).
… You can have a look at my PPT. You will see the homework I gave them. They are all sorts of repetitive exercises. The program for international students is different. That focuses on students’ language skills (Xia Xiao).
For international students, I focus on language skills. I train their listening, speaking, reading and writing skills. Nothing in-depth (Xing Liu).

In contrast to the curriculum implemented for the local “ingroup” students, the teachers collectively imagine a suitable subject content for international students as following the “prescribed textbook” (Jing Lin), focusing on “language skills” (Xia Xiao), choosing “practical” content (Yuan Wang), “repetitive” exercises (Xia Xiao), with an exclusion of profound subject knowledge. The students’ background impacted on these teachers’ decisions on the selection of subject knowledge and content in practice. When teaching the “outgroup” of international students, the teachers enacted a functional or practical “technician” approach. One argument might be that these teachers’ passion for teaching content linked to Chinese heritage does not gain traction with their students in the “outgroup”. Nevertheless, their understanding of self as a CFL teacher of Chinese background seemed certain. That is, the “ingroup” students should not only know the language, but more importantly, be exposed to the heritage of the nation’s classic literature, history and culture and become part of that tradition. The “Chineseness” and the identity of a Chinese language teacher lies in these legacies. This is reflected from some excerpts from the data:

When I teach our own students (local Chinese students), I have the sense of achievement. I feel the things I did are more valuable. You know that the precious knowledge you have accumulated has been passed on to the students. I feel the pride and respectable status as a teacher – 传道授业解惑 (pass on the knowledge, prepare them for a career and solve their puzzles) (Jing Lin).
… (To international students) you feel like you are teaching children sometimes, and the content is very basic, shallow and unchallenging. You lost that kind of proud feeling of being a teacher—a job that is very important and respectable (Wei Le).

These data excerpts portray a shift in their feelings and identity as to what constitutes a successful fulfilling career, with their professional work defined by the need to renegotiate subject knowledge and teaching content in order to engage the “outgroup” students. In response to the necessary content changes, their sense of being a proud Chinese language teacher was lost. They valued the content they taught to local Chinese students as providing them a “sense of achievement” (Jing Lin): pride, dignity and confirmation of the sanctity of their identity as authoritative knowledge holders. The content they chose to teach international students was “basic, shallow, and unchallenging” (Wei Le) due to this group’s lack of foundational heritage knowledge. This finding is comparable with Gong et al.’s (2018) research in which CSL teachers’ objective of teaching was more skill-oriented than knowledge-oriented when facing international students. Even though redefining the curriculum for the “outgroup”, the language teachers maintained their original belief and image of an ideal CFL teacher. The changes they made at a practical level were to better cope with the new conditions or challenge their “outgroup” students had brought. However, their value system around the subject knowledge a CFL teacher should hold and should teach was not changed.

### Hierarchical distant teacher-student relationship versus closely active mutual friendship

The teachers’ relationships with their ‘own’ students (local Chinese students) were different to that established with the international students. Interview data reveals a tendency for the teachers to be serious and strict, showing distance when teaching local students, while engaging with the international students in an approachable, relaxed, and friendly manner:

It’s easier to communicate with international students. Naturally, I feel closer to them and I know them better. A lot of interactions happened with them in my class, but with local students, I feel the distance. They seldom approached me with questions or chitchat (Wei Wang).
In class, interaction happened more with international students, and I was more familiar with them (Xia Xiao).
I personally feel that I have a closer relationship with the international students. It feels like there is an emotional connection and I feel like making friends with them. I am a bit serious with our local students and there is a distance there (Jing Lin).
I do not know why! I am the same teacher, but different when teaching different students. I behave in one way when teaching a class of Chinese students, and another when dealing with a class of international students (Jing Lin).

The participant teachers admitted that they are “the same” teachers but behave “differently” in front of the local and international students (Jing Lin). Through interaction and communication, they established close “emotional connection” with the international students (Jing Lin; Xia Xiao). This relationship came along “naturally” (Wei Wang). However, when with local students they were “serious” and felt “the distance” (Jing Lin; Wei Wang). Most of the teachers ascribed this divergent behavior to differing characteristics within the student cohorts themselves. That is, they saw the change as an accommodating response to ingroup and outgroup differences.

International students mostly actively participate in class activities. They are more willing to speak and interact with me. It does not matter if the content is easy or difficult. They tracked you closely. I can not say my own students are not interested but they normally do not show it on their face. They sometimes do what interests them in class instead of following me (Xia Xiao).
International students comparatively show a more serious attitude and passion to learning. I feel closer with them because they like to share their thoughts with me and make me know better about them. That actually gives me passion to teach them (Wei Le).
They (international students) are interested in the local culture here and they are curious about my daily life. They are keen to get to know me and my life. Some of them like to talk to me about their experience and invite me to join their on-campus activities (Yuan Wang).
They (international students) respect me. They would take the initiative to say hello and goodbye, and they would tell me in advance if they had any needs such as going to the bathroom. When they see me on-campus, they would greet me with a warm hello (Wei Le).
International students like to ask questions in class. They always want to know what this is and why it is so. Maybe this is because this language and culture is totally new and strange to them. They are more curious and eager to learn the knowledge. As for local students, since they have this cultural background, it seems much easier for them to accept the idea and they can understand it just at a glance (Xing Liu).
Emotionally, I am keen on international students because they are always responsive in my class, but I can always expect to get more logic and in-depth answers from local students. We can talk about the latest research in this area and discuss more academic and professional issues. Maybe because of the language barriers, I think it is good to just tell international students the basics and make them clear (Jing Lin).

Data from the interviews indicates that international students as a group tended to show the characteristics of being active, initiative, passionate, willing to connect and express interests, whereas local students tended to be reticent, reserved, stony-faced and indifferent sometimes. The teachers described the international students as “more willing to speak and interact” (Xia Xiao), showing “more serious attitude and passion” about learning, willing “to share their thoughts” and “experience” (Wei Le; Yuan Wang), “curious about” the teacher’s daily life. “take the initiative to greet the teacher” (Wei Le). “More curious and eager to learn the knowledge” (Xing Liu) and “always responsive” in class (Jing Lin). The passion the international students showed for learning, and the explicit interest in and the respect for the teachers all contributed to the teachers’ emotional engagement with this group. For local students, the teachers’ descriptions include “They do not show it (interest in learning) on their face” and “They sometimes do what interests them in class” (Xia Xiao). Although sometimes teachers could “get more logic and in-depth answers” from them (Jing Lin), the teachers did not establish close emotional ties with the ingroup students due to their being less proactive and less expressive in or outside class.

In general, the being and doing of the two groups of students shaped the CFL teachers’ relational identities. They were more rational with the local students and more emotional with their international student cohort. With the local students, they “acted” more as a teacher or an academic. They held tight with the teachers’ “dignity” by being serious in speech and manner. When engaging with their international students, displaying a “natural” and “genuine” persona rather than a more emotionally distant professional figure. This seems comparable with Wang and Du’s (2014) research findings which indicate that CFL teachers reframed their identity toward teacher-student relationship as “equal and plain” with students in the Danish contexts due to institutional impact and “overseas-born status as outsiders” (p. 450). The teachers participating in this study transformed their relational identity with international students. At face value this transformation was not due to the institutional or sociocultural factors but the students’ ethnic and cultural differences only. The behavior of the teachers with the ingroup seems to be very much about being nested in a cultural context – the sociocultural rules implicit in Chinese culture, whereas teaching the outgroup of international students enable the teachers to operate outside of those cultural expectations and experience diverse ways of relating. So even though the teachers viewed the changes in their teaching as being due to the student differences, it was also due to their own (temporary) release from a hegemonic cultural context.

### Sage on the stage versus guide on the side

The teachers described two different modes of teacher pedagogical identity, to some extent related to the two different groups of students Among the six participant teachers, there were four who implemented a teacher-centered or “sage on the stage” pedagogical style whereas the other two comfortably exercised a student-centered or “guide on the side” approach. Thus, the majority of the CFL teachers were expository advocates, as opposed to constructivist practitioners. For the four teachers who practiced expository teaching, they were the center of the teaching and learning, the knowledge holders, the transmitters, and leaders. This was the case especially with the local ingroup students:

The nature of this course is quite theoretical. They rely on my explanations a lot. It will not be effective for them (local Chinese) to learn in a self-directed way (Jing Lin).
For my local students, they prefer to listen to the lecturer’s talk. Discussion in class is not their favorite way of learning. If I make my class a discussion type, most of the students would think: My God (哎呀)! This is a watered-down (很水) lesson (Wei Wang).
Local students in general are reserved. It is hard to make heated discussion happen in class. It often turns out to be the teacher’s one way presentation (Xing Liu).
If I design this lesson as a discussion type (for local students), it means, they need to do a lot of preparation before they come to the class. The problem is they are not used to it. Also, they are under great pressure from their other subjects. I tried this, and it was a failure (Wei Le).
International students are probably used to the mode (teamwork), but for my locals, they might feel teamwork is too much extra work. If it is not allocated to individuals, they feel that it is not their work. Other team members can do it (Jing Lin).

The teachers provided various justifications for their teacher-centered pedagogy with local students. These include the theoretical nature of some subjects thus difficult for student-centered learning, the students’ preference for teacher-directed presentations, students’ views that student-facilitated sessions are “watered-down” teaching, students’ lack of a collaborative learning spirit and increased preparation pressure on students for learner-centered classes. According to these teachers, the local students’ “passive” learning style seemed to be the main factor that contributed to their teacher-centered pedagogical decision. They “rely on the teacher’s explanations a lot” (Jing Lin). “Discussion is not their favorite way of learning” (Wei Wang). They preferred “the teacher’s one way presentation” (Xing Liu). “Teamwork is too much extra work” for them and they were not used to it (Jing Lin). These quotes indicate that the “sage on the stage” approach is needed for the local students primarily. The student expectation of a “sage on the stage” seems to be a cultural construct they bring to the lessons (at least as it is perceived by the teachers).

With reference to the international groups, all four teachers maintained a similar teacher-centered role as a pedagogical approach, however, the evidence for this was based on different concerns:

I focus on training their (international students) language skills. A typical lesson includes my presentation of the content, followed with the exercises I prepared for them to practice. I make sure they master language skills and enhance their communicative competence through lots of repetitive exercises (Wei Wang).
I often ask them questions, but most are close-ended ones as open-ended questions are difficult for them. You saw the questions I left with them today? They can find answers in the textbook. For the final exam I prepared, they can also find ready answers in the textbook (Jing Lin).
This semester, the university combined all my international students into one big class. Ideally, as before I could organize more interaction in class, but now only the first two rows have a chance to be called on, to answer my questions. It is not realistic to look after everybody when the class size is big (Xing Liu).
I applied more teacher-student interaction for this group (international). I did not use much of student-student interaction, and as for student groupwork, there was even less. The interactions were mainly between me and them through close-ended questions (Wei Le).

In response to their “active” international students, the data reflect that the teachers endeavored to create more interactive opportunities in class. However, these so-called “interactions” were basically initiated by the teachers in the form of questions for students to answer. Most notably, the teachers relied on close-ended questions intended to assess students’ content knowledge. There were no data revealing genuine student-student teamwork nor students’ leading activities in the lectures and interviews with these four teachers. It can be argued that the teachers still played a dominant role in the “outgroup” classes. Their pedagogical identity had not changed in response to their more active international students. Those efforts to engage students in one-on-one interactions at most can be acknowledged as expository teaching with constructive characteristics. The necessity of a teacher-centered approach was also due to institutional constraints, in the form of class sizes.

In contrast the other two teachers exhibited a “guide on the side” teaching style across both local and international student groups. They chose not to lecture students but provided opportunities for the students to learn through experiences promoting teamwork. The teachers’ role was to facilitate when needed:

One of my subjects is lexicology for local students. As I said earlier, I did not lecture or teach them. Instead, I uploaded the videos to the forum for them to view. In class, I let them share the information and spend time analyzing language phenomena. I did not give them the answer or solution. They could work out their own answer or draw their own conclusion (Xia Xiao).
I think it is useful to lecture them with knowledge, but this kind of knowledge can be obtained by students themselves. I think teaching them how to analyses and how to think critically is the key for university students (Xia Xiao).
The class for international students is topic-based. I always find out their interested topics. Like last week, I suggested ken lao zu (boomerang children), that did not interest them. I then tried pop music. The discussion was good. Everybody seemed to have something to say or share (Yuan Wang).
When they are doing activities, I will walk around and see whether they need help. Like last time when they discussed about the topic of traffic, one team asked me the hotline service number, I checked and gave it to them. The classroom is their show place. I am just there to help (Yuan Wang).

Rather than positioning themselves as authoritative knowledge holders with a transmissive mission to pass on that knowledge directly to students, these two CFL teachers chose to divert from lecturing, focusing instead on their students’ interests, high level thinking and problem solving. Xia Xiao let the students “share the information and spend time analyzing language phenomena”, and “work out their own answer or draw their own conclusion”. Yuan Wang conducted “topic-based” teaching for students to “have something to say or share” as she said “The classroom is their show place. I am just there to help”. These teachers placed the students at the center of learning, and themselves as facilitators, which demonstrated their constructivist teaching ideology and practice.

## Discussion

The findings from this research have generated some themes worthy of further discussion. Firstly, these Chinese language teachers demonstrated similar social identities or collective identities when engaging with different cross-cultural groups of students, that is, group-based identities. They unanimously felt local students were “their own”, which implies that they belonged to the teachers’ “ingroup”, and international students as “outgroups” were treated differently. Using Hogg’s (2018) argument promoting a social identity perspective, the teachers established “group-based identities” (p. 117). They, as a group of Chinese language teachers, had shared “self-defining attributes”, and had engaged in teaching “to forge an image of what the group stands for and how it is represented and viewed by others” (Hogg, 2018, p. 117). The attributes of this shared identity for the “ingroup” students include their view of the importance of CFL teachers’ subject knowledge, their relational identity when interacting with students, and the majority of the teachers’ pedagogical identity – a teacher-centered expository approach. For the “outgroup” students, the attributes of their shared identities required similarly adjusted views on understandings of subject knowledge, interpersonal relationships, and pedagogical approaches, with simpler curricular values, more relaxed interpersonal relationships, but overlapping pedagogical approaches. These group-based social identities or collective identities, form what can be regarded as, the core identities of Chinese CFL teachers.

In terms of subject knowledge, the relationship between teachers’ self-acknowledged expertise and their identity has been documented (Bryan, 2003; Mulholland and Wallace, 2005; Brooks, 2016) as has the close connection between teachers’ subject knowledge expertise and students’ achievement (Muijs and Reynolds, 2003). In this research, the CFL teachers, as a group, tended to share similar ideas on what and/or how much subject knowledge was required to confirm their identity as successful language teachers. They perceived that a Chinese language teacher should not only be a language expert, but more importantly s/he should be conversant with the nation’s classic literature, historical and cultural knowledge in order to achieve the status of a “dignified” teacher of local student cohorts. When teaching an enriched curriculum beyond language skills to the “ingroup” students, their identity as language teachers was fulfilling and embodied a positive self-image. These attributes were partially maintained during the teaching of the “outgroup” students, although the interview data confirmed the teachers did not have the same sense of fulfilment when presenting a basic, foundational language curriculum required by the international “outgroup” student cohort.

The CFL teachers displayed a dichotomy of identities with regards to their relationships and engagement with the local Chinese and international student groups. With international students, the teachers demonstrated warmth, closeness, and friendliness whereas with the local students they showed distance and strictness. As previous research found, teachers often negotiate their relational identity between “juggling their position as students’ friend or ally” and at the same time “retaining … authority” (Johnston, 2002, p. 103). This may lead to contradictory self-conceptualizations of “who they are” (Akkerman and Meijer, 2011). The co-existence of their contradictory or multiple identities further verifies the argument that “context is one of the significant determiners of the entangled processes of language teacher identity,” and particularly the change to the immediate social context (such as a new group of learners) is very influential in developing teachers’ relational identities (Yazan, 2018, p. 36; Gee, 2000). From the Social Identity Theory perspective, the embodiment of a dichotomous relational identity reflects the teachers’ self-enhancement and attempts at reducing uncertainty (Hogg, 2018). Namely, when engaging with a group of students from unfamiliar cultures, the teachers took on the persona of a “nice” and “friendly” colleague to achieve “positive intergroup distinctiveness” (Hogg, 2018, p. 121). This self-enhancement process is an endeavor to reduce their subjective insecurities brought about by the inclusion of the student “outgroup”. This makes it sound like the teachers were quite instrumentalist in being friendly to the international students. Their comments in the interviews seem to show that they genuinely enjoyed their interactions with the outgroup, and their own sense of relaxation and friendliness emerged as a response to the different cultural characteristics of the students rather than being “instrumentalist” in trying to bring about a sense of inclusion.

In terms of pedagogical identity, the Chinese CFL teachers demonstrated a minor shift within their preferred teaching method. As a group of teachers, the majority maintained expository practice, as the differences in the learning styles or characteristics between the “ingroup” and “outgroup” students were not such that a pedagogical switch resulted. This finding aligns with Han’s (2022) research, which supports the argument that expository teaching dominates China’s higher education system due to historical, social, and cultural reasons. Gong et al. (2021) ascribed the one way transmission style of teaching to Chinese teachers’ “relatively narrower and more ethnocentrically-positioned” identities (p. 13). It can be concluded that the teachers in this research did realize the need and endeavored to teach international students in a more co-constructive way. This finding differed from the results in Yang’s (2019) research, which reported that CFL teachers in the United Kingdom strived to transit to a student-centered teaching approach to adapt English school context even though being hampered by the limited insights of the core values of English education system. This finding is also different from Wang and Du’s (2014) investigation in which CFL teachers in Denmark transformed themselves from “knowledge authoritarian” to “learning facilitators” (p. 446). The inconsistent results indicate that compared to those CFL teachers in overseas contexts, the participant teachers in their own country were more insistent with their original pedagogical identity, with minor shifts to accommodate the two different student groups. The identified minor pedagogical shift when dealing with different groups reflected the teachers’ negotiation between their institutional identity and individual agency. On the one hand, their governing institution may have acted as “laws, rules, traditions, or principles of various sorts” which enabled them to hold “the rights and responsibilities” aligning with their professional identity (Gee, 2000, p. 103). It ensured or even enforced that the teachers’ pedagogical identity strove toward stability and continuity (Akkerman and Meijer, 2011). On the other hand, they mediated the relationship between the new social environment (international “outgroup” students) and their original self (Ruohotie-Lyhty, 2018, p. 25) enacting their “identity-agency” to ensure that any shift was in the range that did not decentralize their pedagogical identity.

## Conclusion

This research examined the identities of a group of CFL teachers through their imagining and conceptualizing of self when teaching students from their own and from other language and cultural backgrounds. Based on teacher interviews, the research concluded that teachers saw student backgrounds and expectations as a determining factor in the development of teachers’ relational identities. However, their value and belief in the importance of the subject knowledge a CFL teacher should possess, and the image of what makes an ideal CFL teacher were not substantially changed across the “ingroup” and “outgroup”. This attribute of their identity was sustained even though the teaching content was modified at a practical level in response to the challenges the new “outgroup” student groups brought. Similarly, when teaching “ingroups” and “outgroups”, the CFL teachers’ pedagogical identity remained stable with only minor modifications, indicating an identity construction that was both stable, and dynamic in response to contextual factors.

This research was conducted through a qualitative method, focusing on a small number of participants from one university. Future studies can consider applying a mixed method to enhance the trustworthiness of the data.

## Data availability statement

The raw data supporting the conclusion of this article will be made available by the authors, without undue reservation.

## Ethics statement

The studies involving human participants were reviewed and approved by Western Sydney University Human Research Ethics Committee. The patients/participants provided their written informed consent to participate in this study.

## Author contributions

HC, WS, and JH contributed to the conception and design of the study. HC collected the data and conducted the literature review. WS performed the data coding and analysis. JH conducted the theoretical analysis and wrote the first draft of the manuscript. QL contributed to the data coding and literature review. All authors contributed to the manuscript revision and approved the submitted version.

## Appendix A: Interview protocol

Can you give some background information about yourself (e.g., age range, qualification, years of teaching etc.)?

Have you had experience of teaching local Chinese students and international students?

How did you feel about yourself as a teacher when facing culturally different cohorts of students?

Did you have to adjust teaching style or method when teaching local and international groups and how does that impact on your pedagogical identity?

Did you have to adjust the way you engage and interact with students when managing local and international groups and how does that impact on who you feel you are as a teacher?

What is your imagination or criteria of being an ideal CFL teacher in terms of the subject knowledge a teacher should be equipped?

How is this reflected when you face local and international groups?
